# Validation of the Chinese version of the resilience scale for the oldest-old

**DOI:** 10.3389/fpsyg.2023.1055301

**Published:** 2023-02-17

**Authors:** Ning Wang, Hongyu Chen, Minyi Zhang, Yuanyuan Wang, Zhihan Xue, Xixi Hao, Yan Liu

**Affiliations:** ^1^Department of Neurosurgery, The First Affiliated Hospital of China Medical University, Shenyang, China; ^2^Department of Nursing, Children’s Hospital of Nanjing Medical University, Nanjing, China

**Keywords:** RSO, reliability and validity, Chinese version, oldest-old, resilience, RSO

## Abstract

**Background:**

Resilience is one of the most important variables associated with adaptive ability. The resilience scale for the oldest-old age (RSO) has been designed to measure the resilience among the oldest-old people. Originally developed in Japan, this scale has not been used in China. The objective of this study was to translate the RSO into Chinese and investigate its validity and reliability among the community’s oldest-old adults aged ≥80 years.

**Methods:**

A total of 473 oldest-old people who came from communities were recruited by convenience sampling for the assessment of construct validity using exploratory factor analysis (EFA) and confirmatory factor analysis (CFA). In addition, internal consistency reliability, test–retest reliability, face validity, and content validity were used to evaluate the psychometric characteristics of RSO.

**Results:**

The RSO demonstrated good face validity and content validity. The content validity index of the Chinese version of the RSO was 0.890. Moreover, one factor was extracted by exploratory factor analysis, which accounted for 61.26% of the variance. The RSO had high internal consistency with a Cronbach’s alpha = 0.927. The test–retest reliability was 0.785. The item-total correlations ranged from 0.752 to 0.832.

**Conclusion:**

The results of the study indicate that the Chinese version of the RSO questionnaire has good reliability and validity and can be recommended for use by health and social service agencies as a method for assessing the resilience of the oldest-old in the community.

## Implications for practice

The validated Chinese version of the RSO is an available and reliable tool for evaluating the resilience of the oldest-old people in China, suggesting significant clinical application value.

## Introduction

Aging is a major social issue all over the world. For decades, the population of older people aged 60 and over has been growing in almost every country in the world (Ju et al., 2018). Similar to global trends, China’s population has aged rapidly in recent decades (Zhang et al., 2017). In 2019, the Chinese population constituted 18% of the world’s population, with 164.5 million Chinese citizens aged 65 and above (65+), and 26 million aged 80 or above (80+). By 2050, there are expected to be 1.4 billion Chinese, with 365 million people over the age of 65, accounting for 26.1% of the country’s total population (Fang et al., 2020). According to the world social standard of aging (in the total population, over 60 years old (>10%) and over 65 years old (>7%), China is in the rapid developmental stage of population aging (Luo et al., 2021). In addition to the rapid expansion of the elderly, the proportion of oldest-old people has increased significantly. According to authoritative forecasts by the United Nations Department of Economic and Social Affairs, by 2050, the number of people aged 80 and above in China will exceed 100 million, accounting for 30% of the elderly population, which presents the country with many challenges in dealing with the problems caused by aging (Xu et al., 2022). Because the population continues to live longer, issues such as poor physical health and mental health will be more prominent in the older population, who face more challenges than any other group; here, adapting to the changes and losses associated with aging is crucial for older people. However, it is commonly agreed that resilience contributes to well-being and quality of life when confronting adversities, and hence may be a key resource for aging well (Siltanen et al., 2021). Studies have shown that resilience is a fundamental factor for adapting to aging and for aging well (Hayman et al., 2017). With more challenges to face than any other segment of the population, it is crucial to explore the sources of resilience for aging and older adults (Manning et al., 2019).

Resilience has been broadly defined as the human capacity to adapt to tragedy, trauma, adversity, hardship, and major stressors (Wermelinger Ávila et al., 2017). Itzhaki et al. (2015) and Prosser et al. (2017) acknowledged that resilience has not been consistently defined in the literature and that, conceptually, it has ranged from an individual quality and trait to a process. Over the past two decades, the concept of resilience has significantly changed from a trait-oriented to an outcome- or process-oriented approach (Chmitorz et al., 2018). When conceptualized as a personality trait, resilience moderates the negative effects of stress, promotes adaptation, and enables individuals to positively face adversity. In recent years, resilience is increasingly considered as an outcome (outcome-oriented approach), meaning that mental (or physical) health is maintained or regained despite significant stress or adversity (i.e., short-term/acute or long-term/chronic, social or physical stressors; Kalisch et al., 2015). The current evidence supports resilience as a multifactorial process that interacts and accumulates throughout life, involving individual aspects, environmental context, quantity and quality of life events, and the presence of protective factors.

A developmental approach to resilience suggests that when people of different ages face the same situation, they may have different experiences of stress (Hayman et al., 2017). This concept has primarily been studied in young adults, but currently, a growing area of research has focused on the role of older adults and resilience in successful aging (MacLeod et al., 2016). It has been shown that the psychological resilience of the oldest-old and adults differs in terms of risk factors, protective factors, and resilience outcomes (Manning et al., 2016; Browne-Yung et al., 2017; Hayman et al., 2017). Some of these differences include the following: (1) The risk factors for adult psychological resilience are not significantly influenced by age and come mainly from posttraumatic growth, anxiety, and depression. The age-related adversities faced by oldest-old people, such as an age-related decline in organ function and chronic illnesses, the changing social roles of older people after retirement, and a reduced sense of self-worth, exacerbate anxiety and depression, posing a severe threat to their mental well-being. (2) Protective factors, which are known as assets, resources, or strengths, play a role in achieving resilience. The protective factors of psychological resilience in adults are mainly derived from personal qualities, personal capabilities, and social support. In times of adversity, older people can look back at their unique historical experiences to gain insight and wisdom. (3) Resilience outcomes differ: health outcomes for the oldest-old in terms of psychological resilience include both physical and psychological outcomes.The main indicators of psychological outcomes are well-being and self-perceptions of successful aging. Adults focus on health promotion and the ability to recover from illness.

In older persons, resilience has been defined as the ability to achieve, maintain, and recover physical or emotional health after diseases and losses (Hardy et al., 2004; Fontes and Neri, 2015). With an increasingly aging population facing age-related adversity at an unprecedented rate, an individual’s ability to demonstrate resilience has been linked to reduced risk of depression and mortality, which is becoming increasingly important for older adults, caregivers, and clinicians (MacLeod et al., 2016; Cosco et al., 2017). The oldest-old possess a variety of sources of resilience that help them adapt well to stress and loss. Research has confirmed that resilience in older people may be related to general levels of health and well-being, with those with greater health and well-being being more able to cope with adversity (Tay and Lim, 2020). In addition, positive personality factors have been shown to moderate the impact of poor health on subjective well-being, so that some older people may be naturally more resilient than others. Religious and spiritual beliefs have also been shown to promote psychological resilience in older people (Manning and Miles, 2018).In addition to internal factors such as personalities and religious beliefs, external factors such as family support, levels of social interaction, and early life conditions are also vital to the resilience of older people (Li and Ow, 2022). With better resilience, older adults can compensate for lost functional capacity and physical health, with better health outcomes, such as successful aging, less depression, and a longer life (MacLeod et al., 2016).

Psychological resilience assessment tools are a fundamental approach to psychological resilience research. Therefore, it is essential to develop reliable and valid tools to assess the resilience of the oldest-old people, enhancing their resilience to adversity and improving the quality of life of older adults. Some studies have shown that psychological resilience scales such as the BRCS, CD-RISC, RSOA, and RS are applicable to older people (Ho et al., 2012; Cosco et al., 2016; Li and Ow, 2022).

Among the scales, the CD-RISC was developed by Connor and Davidson (2003) to measure psychological resilience as a personality trait. It consists of 25 items, including five dimensions: personal competence, tolerance of negative feelings, acceptance of change, control, and spirituality. The Cronbach’s alpha coefficient was 0.89, and the remeasurement reliability was 0.87. It is widely used by scholars in different countries and has been adapted to produce a scale of confidence and validity that is more appropriate for the region. For example, Campbell-Sills and Stein (2007) compiled a short version of the 10-item CD-RISC10-items based on the original scale. The CD-RISC-10 has been shown to have good psychometric properties in older adults (Cronbach’s α = 0.89; Rezaeipandari et al., 2022). The RS was developed by Wagnild and Young (1993) based on a study of the psychological status of bereaved older women as a way to measure individual traits that enhance an individual’s ease of adaptation to stress; the scale, consists of 25 items (referred to as RS-25) on five dimensions: perseverance, self-confidence, meaningful life experiences, sense of ease, and composure. The Cronbach’s alpha coefficient for the RS-25 was found to be 0.90, and the correlation between the items and the scale ranged from 0.37 to 0.75. Yang and Wen (2015) demonstrated through a cross-sectional study of 498 older adults that the Chinese version of the RS is reliable for measuring psychological flexibility in older Chinese adults. The BRCS was developed based on a sample of people with rheumatoid arthritis (Sinclair and Wallston, 2004). The scale consists of four items designed to capture the tendency to cope with stress in a highly adaptive manner and has been validated for measurement invariance in Peruvian and Spanish elderly people (Tomas et al., 2021). The RSOA, a psychological resilience scale specifically adapted to older adults, was developed by Taiwanese-Chinese psychologist Yang-Tzu Li (Li and Ow, 2022). The scale consists of four dimensions, which are related to the life satisfaction of older people, factors with 15 items.

Although these scales have previously shown favorable psychological properties in their use in populations of older individuals, they have limitations. For example, resilience may be expressed differently in people facing different types of life adversity (Li and Ow, 2022). In addition, these scales were not originally developed for the oldest-old and, therefore, may be limited in assessing the psychological resilience of the oldest-old. In addition, the use of reliable and valid as well as practical assessment tools can also help scientifically and objectively assess the psychological resilience of the oldest-old people while improving their ability to cope with stress, thereby contributing to a better quality of life.

In the absence of a questionnaire-based instrument to measure the psychological resilience of the oldest-old, Akatsuka and Tadaka (2021) developed the Japanese version of the RSO, a one-dimensional validated instrument consisting of nine items to assess the psychological resilience of the oldest-old. In this study, older adults were interviewed; the interview results were summarized, scale entries were extracted, and an exploratory factor analysis was conducted to obtain one factor. The questionnaire has been validated and is relevant to the oldest-old population, demonstrating excellent psychometric properties. The total scale has a Cronbach’s alpha of 0.800 in the Japanese context and is considered an adequate, reliable, and valid scale to effectively assess the adaptive capacity of the oldest-old for age-related changes and losses (Akatsuka and Tadaka, 2021).In addition, this scale is short and easy to complete and has not been translated for use in other languages. Research on the psychological resilience of older people in China is relatively recent, and research on the psychological resilience of older people is still in the exploratory stage. A variety of scales and instruments measuring resilience are currently available and have been tested in different populations in China (Xie et al., 2016; Wu et al., 2017; Gao et al., 2019; Chen et al., 2020; Cheng et al., 2020; Shi et al., 2021; Tian et al., 2021).

However, there is no validated psychological resilience scale for the oldest-old. The present study aimed to translate the RSO into Chinese, develop a culturally adapted version of the RSO for the Chinese context, and investigate its validity and reliability among the oldest-old in the Chinese community. It was assumed that the Chinese version of the RSO would have the same factor structure as the original scale. This can provide a valid measure for assessing psychological resilience in nursing practice.

## Methods

### Study design

The present study used a cross-sectional design and collected data from October 2021 to June 2022. Data were collected from a convenience sample of the oldest elderly living in communities in Liaoning Province, northeast China. The participants were mainly recruited from three elderly activity centers (Ba Yi Community, Wen Cui Community, and Duo Fu Community) in Shenyang, Liaoning Province, to increase the representativeness of the sample. According to international questionnaire design and psychometric principles (Marsh et al., 1998), the sample size should be 5–10 times the number of items and should be expanded by at least 20% to ensure an adequate sample size. The sample sizes for the exploratory factor analysis and confirmatory factor analysis (CFA) norms were 100 and 200, respectively. Firstly, a sample size of at least 108 was calculated because the number of entries in the RSO was 9. The analysis ultimately included 473 participants. The study was approved by the Institutional Review Board of the First Hospital of China Medical University (approval number: 2021[435]).

### Participants and data collection

The inclusion criteria were as follows: (1) people≥80 years old; (2) those with the ability to communicate; (3) those in a stable condition; and (4) those who had provided signed informed consent. People who were not permanent residents of Shenyang or who suffered from a severe or acute disease were excluded.

Prior to participating in the study, the participants were informed of the purpose of the study and their right to withdraw. The survey was conducted at a community health center where community staff invited the oldest-old to participate in the survey by contacting the community health centers through WeChat— a mobile application that allows you to chat by sending text, pictures, voice, video, and so forth— Older People’s Group and by telephone. During the survey, compliance was improved by distributing small gifts or some simple physical examinations such as vital signs, height, and weight. Considering the potential literacy and visual limitations of the oldest-old, the survey was conducted using face-to-face interviews and a structured questionnaire. The questionnaire was administered by a surveyor who read the questions aloud, item by item, following uniform guidelines, with interpretation by a trained research nurse, when necessary, and completed on behalf of the oldest-old according to their true wishes. In total, 473 (227 male, 246 female) older adults aged 80–102 years (mean = 84.30 years, standard deviation = 4.00) volunteered to participate in a face-to-face interview conducted by trained researchers to assess the psychological status of the oldest-old.

### Instruments

#### Socio-demographic and disease-related information sheet

A structured questionnaire was designed to collect demographic data on older people, including age, gender, marital status, and religion. There were also several disease-related characteristics, such as type and type of chronic disease.

#### The Chinese version of the resilience scale for oldest-old people

The resilience scale for the oldest-old age (RSO) was originally developed in Japan to measure resilience in the oldest-old people. The initial 20-item scale was validated and finally revised to a nine-item unidimensional scale. The validated factor analysis model fitted the scale well, with loadings of 0.476 to 0.760 for each item on its dimension, all >0.40, and with no double loading. The self-administered instrument used a 4-point Likert scale ranging from 0 (disagree) to 3 (agree). The total score ranges from 0 to 27, with higher scores indicating better resilience.

### Translation and cross-cultural aspects of the resilience scale for oldest-old people

With the permission of Eiki Akatsuka, the author of the original RSO scale, the scale was translated and culturally adapted in accordance with the guidelines recommended by the American Academy of Orthopedic Surgeons Evidence Based Medicine Committee (AAOS).

Phase I consisted of four steps. (1) In forward translation, two translators (one professor with a PhD in nursing overseas and one associate professor in Japanese at a university) who were native Chinese speakers and proficient in Japanese translated the scales independently and obtained two translated versions. (2) In the step of synthesis, a third bilingual and bicultural translator compared the two translations with the original scale and organized a discussion between the translator and research team to reach an agreement on the initial translation. The translators and research team discussed and agreed on the translation version, and the initial translation version was formed. (3) Next, the initial translation was back-translated by two native Japanese translators who did not know the original scale, and two back-translated versions were obtained. (4) Finally, in the step of cultural adaptation, five experts (including an associate professor in Japanese, a professor in epidemiology and public health statistics, an associate professor in psychometrics, a chief physician in clinical geriatrics, and an associate professor in psychological nursing; one master’s degree and four doctorates) were invited to compare the guidelines, entries, and responses of the composite version and original scale, and to make an equivalence between the original scale and Chinese version of the first draft scale. The rhetoric was modified to take into account the experts’ opinions and Chinese national conditions: Item 2 was changed from “I can accept the aging of my body and be open to it” to “I’m comfortable with my body aging;” Item 4 was changed from “For me, being around friends gives me energy” to “The harmony with the friends around me brings me vitality;” Item 8 was changed from “I want to see the world change in the future” to “I hope to witness the changes in society in the future.” These changes were made to be more in line with the expression habits of the Chinese elderly, to be easy to understand, and to have a better organized version 3.

Phase II consisted of two steps (1) First, in the pilot study. Firstly, after numbering each of the seven administrative districts in Shenyang, one administrative district (Huang Gu District) was randomly selected by lottery. Then two community health centers— Bayi Community and Nanhu Community— were randomly selected from the communities within the jurisdiction of Huang Gu District. In June 2022, a small-scale presurvey was conducted with 30 elderly community people who met the inclusion criteria, and a brief one-on-one interview was conducted with 10 patients to assess whether the language expression of the scale was clear and easy to understand, and to find whether the elderly people had questions and suggestions about the content of the scale. Based on the feedback, the language expression of the entries was adjusted and further revised to be more easily understood by our elderly people. Regarding the language, the Chinese version of the RSO was clear and consistent with the semantic meaning of the original scale. The formulation of the two entries was amended as follows: Item 6 “Even if things do not go as well now as they did when I was younger, I do not feel bothered was amended” to “Even if things do not go as well now as they did when I was younger, I do not feel sullen;” Item 9 “Getting older is not as scary as I thought” was amended to “Getting older is better than I thought.” (2) In the next step, Professor Eiki Akatsuka, the original author of the RSO scale, was invited to proofread the consistency of the Japanese back-translation scale with the original scale, because some words and phrases could not be translated literally because of the semantic differences between the two languages. Item 2 was changed from “I’m comfortable with my body aging” to “I can handle the aging of my body very well:” Item 3 was changed from “I know the place where I live very well” to “I feel a sense of belonging where I live;” Item 4 was adjusted from “The harmony with the friends around me brings me vitality” to “Getting along with the people in my area gives me energy.” The Chinese version of the RSO was created after the above steps had been completed, resulting in a nine-item questionnaire that was consistent with the number of entries in the original scale.

## Statistical analysis

All analyses were performed using SPSS 26.0 and AMOS 24.0 for Windows versions. Statistical significance was set at *p* < 0.05 (two sided). A descriptive statistical method has been used to describe the personal characteristics of participants. Qualitative variables are expressed as counts (N) and percentages (%). Quantitative variables are expressed as the mean and standard deviation (SD). Reliability refers to the degree of consistency and stability of the measurement results of a research instrument. Commonly used indicators include internal consistency and retest reliability (Feenstra et al., 2020); validity was evaluated by Content Validity Index (CVI) and structural validity.

The aim of item analysis for item discrimination tests is to determine the validity and appropriateness of the questionnaire items. A couple of discrimination tests were used: (1) First, we used the critical ratio method, in which 473 participants were divided into two groups by dividing the Chinese version of the RSO into 27% before and 27% after the cut-off point, and an independent sample t-test was performed to compare the differences between the high and low groups. The scale entries with statistically insignificant differences were removed after item analysis (*p* ≥ 0.05). (2) We also performed a homogeneity test. Here, the correlation and consistency between the measured attributes of each item of the scale and the total scale were assessed using the correlation coefficient method. The correlation coefficient between each item and the total score of the scale was calculated, and those items with very low correlation coefficients (r < 0.3) were deleted. A correlation coefficient of 0.4 to 1.0 indicates the good correlation and overall differentiation of entries, while a correlation coefficient of <0.4 indicates a poor representation of entries and should be removed.

The review panel consisted of seven experts from hospitals, schools, and the community, all with senior titles or master’s degrees or above and who had extensive experience in geriatric care and geriatric psychology research. The panel members had 1–22 (18.30 ± 2.30) years of experience, were familiar with knowledge of geriatric care and, the development of measurement tools and psychometric characteristics, and understood the content of the scales. Because the oldest elderly participants in the pretest reported that the items were clear and understandable and had no difficulty in completing them, the experts considered the scale to be comprehensive. They used a Likert scale of 4 to assess the representativeness, logic, and relevance of the items to the scale topics, with 1 = not relevant, 2 = somewhat relevant, 3 = relevant, and 4 = very relevant (Polit and Beck, 2006). The CVI was calculated to estimate content validity at the item level (I-CVI) and the scale level (S-CVI). The I-CVI is the result of dividing the number of experts giving a rating of 3 or 4 by the total number of experts, the scale’s content Validity Index (S-CVI) is calculated as the average of the Item Content Validity Index (I-CVI) for all items in the RSO (Luo et al., 2021). The content validity index was rated as acceptable when the I-CVI and S-CVI/Ave were at least 0.78 and 0.90, respectively (Almanasreh et al., 2019).

Construct validity reflects the correspondence between the scale’s measurement entries and measurement dimensions (Clark and Watson, 2019). Construct validity was assessed using exploratory and confirmatory factor analysis. From the 473 valid questionnaires, the total sample was randomly divided into two groups using the simple random method. Exploratory factor analysis was performed in 236 cases (group A). Confirmatory factor analysis was performed in 237 cases (group B). The exploratory factor analysis (EFA) was conducted using principal component analysis (PCA) and a maximum variance scheme to determine the number of common factors with eigenvalues (≥1) and factor loadings >0.4 as the attribution criterion, and to consider deleting entries with multiple loadings with factor loadings <0.4. CFA was conducted to test the validity of this construct by building a model based on the original data and verifying the fit of the structural model to the actual data based on the standard path coefficient plots and various fit indices of the model. The ideal fitting index of the confirmatory factor model was as follows: the ratio of Chi-Square to its degrees of freedom ($\chi^{2}$ /df) < 3; Comparative Fit Index (CFI), Incremental Fit Index (IFI), Goodness of Fit Index (GFI), and Tucker-Lewis Index (TLI) were all >0.9. The root mean square error of approximation (RMSEA) and root mean square residual (RMR) were both <0.008; the standardized root mean residual (SRMR) was < 0.10.

Thirty elderly people willing to be measured again in 2 weeks were selected, and their telephone numbers were kept. After 2 weeks, the reliability of the retest was assessed. Test–retest reliability can reflect the stability of the measurement results. Stability was assessed through an intraclass correlation coefficient (ICC) analysis using a two-way random effect model. The ICC coefficient at the 95% confidence level was calculated using a two-way mixed model to measure the retest reliability. It is generally accepted that when the ICC is >0.70, the consistency between the two measurements is good, and the reliability of the scale is high, with an ICC between 0.3 and 0.7 indicating moderate and an ICC < 0.3 indicating weak reliability (Aaronson et al., 2002; Terwee et al., 2007; Hervé et al., 2019).

The internal consistency of the items that comprising the RSO was assessed using Cronbach’s alpha coefficient, which is evaluated as adequate if it is at least 0.70 (Cosco et al., 2016; Alvariza et al., 2018). Pearson’s correlation was used to assess the item-total correlations.

When a questionnaire or scale is used to test a potential trait or ability of a test subject, the stability of the items is examined and assessed based on the results of the test, which is known as Differential Item Functioning (DIF; Garcia et al., 2021). The presence of DIF in questionnaire items may lead to measurement bias, resulting in the same test being biased toward different groups (Jones, 2019). Therefore, it is important to test the scale items for DIF. An iterative hybrid of ordinal logistic regression and item response theory (IRT) was used to detect gender-related DIF, with a change in the McFadden pseudo R^2^ above of 0.02 as the DIF criterion (Choi et al., 2011). We used the lordif package version 0.3–3 (Choi et al., 2011) in R (R Core Team, 2016) to carry out the DIF analysis.

## Results

### Participant characteristics

The participants’ characteristics are presented in Table 1. A total of 473 participants aged 80 years and over were recruited of which 227 were men (48.0%), and 246 were women (52.0%). The median age of the 473 participants was 69.0 years (range of 80 to 102 years), and the mean ± standard deviation was 84.3 ± 4.0 years. Nearly half of the participants were married (51.0%). The details are provided in Table 1.

**Table 1:** Social and demographic information of the participants.

		(*n* = 473)	
		Number or Mean ± SD	(%) or (Range)
Age, years		84.3 ± 4.0	(80–102)
	80–84	234	49.4
	85–89	204	43.1
	90–95	25	5.3
	95–99	5	1.1
	≥100	5	1.1
Sex	Female	246	52.0
	Male	227	48.0
Educational level			
	Primary school or below	145	30.7
	Secondary school	134	28.3
	High school	120	25.4
	College degree and above	74	15.6
Marital status			
	Unmarried	20	4.2
	Married	241	51.0
	Divorced	49	10.4
	Widowed	163	34.4
Religion	None	230	48.6
	Buddhist	110	23.2
	Taoist	5	1.1
	Christian	90	19.0
	Muslim	14	3.0
	Catholic	24	5.1
Chronic diseases		1.9 ± 0.9	(0–3.0)
	Hypertension	206	43.6
	Diabetes	97	20.5
	Stroke	110	23.2
	Cardiovascular disease	27	5.7
	Cataract	27	5.7
	Osteoporosis	6	1.3

SD, standard deviation.

### Item analysis

The differences between the mean scores of the 27% high and low groups were compared using independent sample *t*-tests. The results showed a significant difference between the scores of all entries in the high and low groups (*t* = 12.85–25.25, df = 275, *p* < 0.01), indicating that the Chinese version of the RSO entries was of good quality and had good discriminatory power. Correlation coefficient analysis showed positive correlations between the items and total scores, with Pearson correlation coefficients ranging from 0.752 to 0.832 (r > 0.4), all of which were statistically significant (*p* < 0.001), indicating good discrimination and representativeness of the items (Aaronson et al., 2002); therefore, all items were retained (see Table 2).

**Table 2:** Item analysis (*n* = 473).

RSO items	Mean	SD	Extreme group comparison	Item-total correlations		Homogeneity test
			Criterial ratio (CR)	Item-total correlation	Adjusted item-total correlation	Cronbach’s α if item deleted
Q1	2.21	0.799	23.996	0.801**	0.747	0.918
Q2	2.02	0.899	28.586	0.799**	0.736	0.918
Q3	2.37	0.831	23.062	0.813**	0.759	0.917
Q4	2.19	0.899	30.102	0.805**	0.744	0.918
Q5	2.31	0.847	24.741	0.832**	0.782	0.915
Q6	1.94	0.858	32.134	0.780**	0.716	0.920
Q7	2.16	0.854	19.797	0.752**	0.681	0.922
Q8	2.26	0.861	24.266	0.815**	0.759	0.917
Q9	1.89	0.918	25.970	0.760**	0.685	0.922
Standard			≥3.000	0.400	0.400	0.927^a^

** *p* < 0.01.

^a^The Cronbach’s α of the RSO was 0.927.

### Reliability test

Cronbach’s α and the item-total correlations were calculated to examine the internal consistency of RSO. The Cronbach’s α coefficients of the total scale were 0.927, indicating adequate internal consistency (Terwee et al., 2007; see Table 2). The result estimated from the ICC was 0.76 (*p* < 0.05), indicating acceptable stability of the instrument (Aaronson et al., 2002).

### Validity testing

#### Face validity and content validity

To assess the face validity, the RSO questionnaire was administered to nine senior citizens to examine how they perceived and interpreted the items. The participants reported that the RSO was clearly worded and that they had little difficulty understanding it. The content validity of the scale was evaluated using the item and scale-level CVIS. The results show that the content validity index of all items of the scale was above 0.86 and that the content validity index of the total scale was 0.90, indicating that the content validity of the scale was good.

#### Construct validity

An exploratory factor analysis using SPSS 25.0 was performed on the first sample using principal component analysis. The Bartlett’s spherical test for the Chinese RSO scale reached a significant level (χ2 = 1303.225, *p* < 0.01) and the KMO value was 0.911, indicating that the scale was suitable for exploratory factor analysis.

In the principal component analysis of EFA, we applied Velicer’s minimum average partial (MAP) test and parallel tests to confirm the number of factors (Ye et al., 2018a). In the parallel tests, the raw eigenvalues, sampled eigenvalues, and percentage eigenvalues continued to decrease as the RSO fixation factor increased. When the fixation factor is 1, the raw eigenvalues are much higher than the sampled eigenvalues and percentage eigenvalues. When the fixed factor is 2, the raw eigenvalues are smaller than the sampled eigenvalues and percentage eigenvalues. Therefore, 2 is out of the range of the most appropriate factors, so 1 is the recommended maximum number of factors. At this point, the maximum amount of variation in RSO can be accounted (see Table 3). The MAP tests also showed the smallest average 4th-power partial correlation of 0.0014 when the root was 1 (see Table 4). The cumulative variance contribution of this factor was 61.261%, indicating that the extracted common factor had strong explanatory power for the dimension to which it belonged, its structural validity was good, and the information content of the study items could be effectively extracted. The loadings of each item in the factor were > 0.4 (all *p* < 0.001), indicating that there were no items that needed to be removed and that validation factor analysis could be conducted (see Table 5).

**Table 3:** List of RSO parallel test eigenvalues and eigenfactors.

Root	Row data	Means	Percentiles
1.00	5.51347	1.30811	1.40102
2.00	0.73473	1.20292	1.26987
3.00	0.66688	1.12452	1.18256
4.00	0.53472	1.05553	1.10296
5.00	0.40902	0.99127	1.03399
6.00	0.37501	0.93021	0.97485
7.00	0.30856	0.86685	0.91603
8.00	0.24493	0.80004	0.85496
9.00	0.21269	0.72056	0.78370

**Table 4:** Velicer minimum average partial (MAP) test.

Root	Average part r sq	Average part r sq
0.00	0.32191	0.11095
1.00	0.03675	0.00251
2.00	0.05256	0.00531
3.00	0.07795	0.01693
4.00	0.11265	0.05896
5.00	0.18383	0.08575
6.00	0.25689	0.14159
7.00	0.45407	0.33722
8.00	1.00000	1.00000

**Table 5:** Results of the exploratory factor analysis of RSO.

No.	(*N* = 236)	
	Item	Loading
1	I’m enthusiastically living my life every day. 我每天都干劲满满, 积极生活	0.819
2	I’m dealing with my physical decline well. 我可以很好地应对身体的衰老	0.772
3	I feel attached to the area I live in. 我对自己居住的地方有归属感	0.824
4	Spending time with local residents from my area cheers me up 与我所居住地区居民的融洽相处给我带来活力	0.776
5	I clearly express my thoughts and feeling to others. 我可以清楚地表达自己的感受和意见	0.821
6	I do not get bothered by something that does not go as well as before 即使现在不会像年轻时那样事事顺利, 我也不会感觉烦恼	0.771
7	I have things that I do to maintain my health condition as much as possible 为了保持现在健康的身心状态, 我做出了相应努力	0.731
8	I would like to see more of how the world will turn out to be in the future. 我希望见证未来社会的变化	0.818
9	Getting old is not as bad as I used to think. 年龄增长比我想象中要更好	0.704
Eigenvalue		5.513

Principal factor analysis with non-rotation.

**Figure 1:**
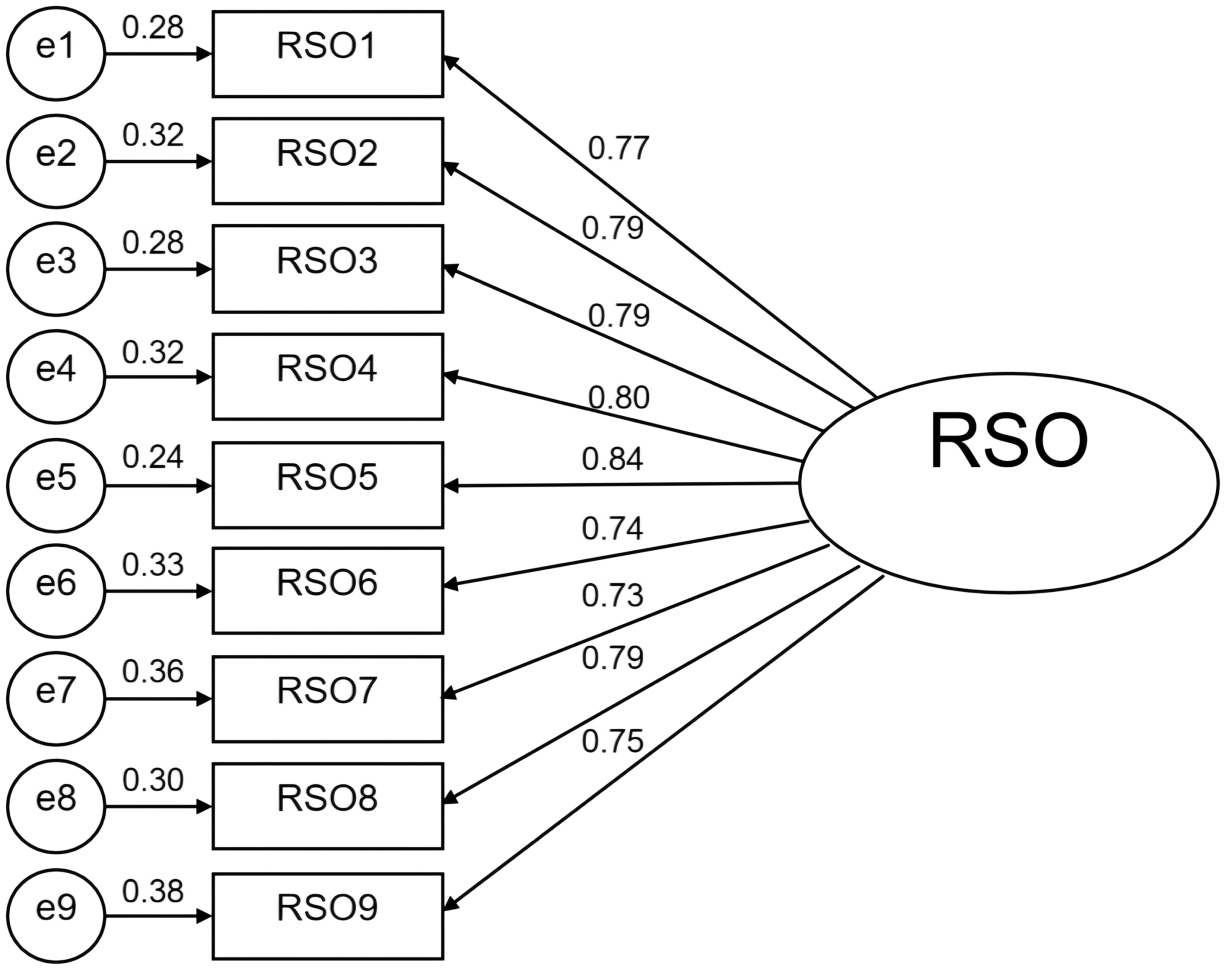
Results of the confirmatory factor analysis for RSO including a single factor.

The internal structure of the scale was explored using AMOS 24.0 to obtain a one-factor structural model. The results of the validation factor analysis were (χ2/df) = 2.206 and root mean squared error of approximation (RMSEA) = 0.071, which met the test criteria, indicating that the model was reliable and applicable. The other model fit indices, the normative fit index (NFI), value-added fit index (IFI), Tucker-Lewis fit index (TLI), and the comparative fit index (CFI), were all in the range of 0.9–1.0 (see Table 6). All the fit indicators met the statistical criteria, indicating that the one-factor structural model fit well and had good structural validity (see Figure 1).

**Table 6:** Fit indices of the models.

Fitting INDEX	*x*^2^/df	RMSEA	GFI	AGFI	NFI	TLI	CFI	IFI
Fitting standard	<3.00	<0.08	>0.90					
modified result	2.206	0.071	0.945	0.909	0.958	0.968	0.976	0.977

*x*^2^/df, chi-square/degree of freedom; RMSEA, root mean square error of approximation; GFI, goodness of fit index; AGFI, adjusted goodness of fit index; NFI, normed fit index; TLI, Tucker-Lewis fit index; CFI, comparative fit Index; IFI, incremental fit index.

### Gender-related differential item functioning analysis

The results in Table 7 indicate that no items were identified as differential item functioning on this unidimensional scale. That is, the level of traits measured on this scale was the same for both male and female subjects.

**Table 7:** Results of functional differences in items on gender.

Item	(Gender)
	McFadden’s R^2^
1	0.0017
2	0.0003
3	0.0067
4	0.0003
5	0.0006
6	0.0045
7	0.0009
8	0.0003
9	0.0004

## Discussion

Resilience scale for the oldest-old age measures the resilience in the oldest-old adults. It is a brief self-reported tool that is not time consuming, and the instructions for use are brief. In addition, it is easy to calculate the score. Use of the RSO for oldest-old individuals can help support professionals to assess the resilience among this group and inform an individualized support approach. In addition, by explaining professional evaluations in individuals, the oldest-old can be made aware of their strengths or weaknesses related to adaptation to aging. Following rigorous and systematic guidelines, the present study aimed to translate the Japanese version of the RSO into Chinese and complete language validation and psychometric examination in the oldest elderly population in China.

Our results show that the RSO had good validity and reliability among the Chinese oldest-old people, supporting its clinical application in measuring resilience. These results coincide with the original version (Akatsuka and Tadaka, 2021). The scale identifies entries through item analysis and is the basis for accurate reliability analysis. The results of the item analysis show that the scale had sound measurement properties, which indicates it had acceptable entry homogeneity and acceptable discrimination and could effectively reflect the degree of psychological resilience of older people. In addition, the test–retest reliability over a 2-week period showed acceptable stability over time (ICC = 0.93).

The present study examined the validity of the scale in two areas: content validity and construct validity. Content validity reflects the extent to which the actual content of the scale is relevant to the content to be measured. In addition, seven clinical, nursing, and psychological experts in the geriatric field were invited to assess the content validity of the Chinese version of the RSO scale by means of expert consultation. The results of the present study showed that the I-CVI was 0.86 and the S-CVI was 0.90. The Chinese version of the RSO scale had good content validity, suggesting that the translated items well reflected the concepts measured and were readable. A combination of exploratory and confirmatory factor analyses was used to analyze the construct validity of the Chinese version of the RSO. Both the EFA and CFA met the measurement properties, together confirming that the scale had a one-factor structure, indicating that the scale had sound construct validity.

Internal consistency reflects the interrelatedness of items on a scale and is a measure of whether all items assess the same construct (Zhou et al., 2021). It is generally accepted that the higher the reliability, the higher the consistency and stability of the scale. If the Cronbach’s alpha coefficient is >0.800, the reliability of the scale is high. The results of the study have shown that the Cronbach’s alpha coefficient of the Chinese version of the RSO was 0.927 (>0.8), indicating that the scale had good internal consistency; hence, the items can well reflect the psychological resilience of older adults in a relatively consistent manner.

The quality of a scale depends on its reliability and validity. The translation of an existing instrument into another language and/or its application to another cultural group requires considering the impact of cultural adaptation on its reliability and validity because the vocabulary, language logic, selection settings, and so forth of a scale vary from one culture to another (Hodiamont et al., 2021). Through a rigorous process of translation, back-translation, expert consultation, and pretesting of the scales, the Chinese version of the scale was fully guaranteed to be equivalent to the original scale. In the present study, the back-translated version was sent to the original authors for proofreading, and the authors of the source scales were contacted several times to confirm the translation. This was mainly done because the original authors had more authoritative rights to modify the scale, which could ensure the maximum semantic equivalence and scientific rigor of the Chinese version of the scale, enhance the robustness of the translation and linguistic validation, and promote the application and cooperation of the scale. However, the shortcoming is that the source scale authors may not be able to communicate more fully with the researchers when giving feedback on controversial translation issues because of language limitations. As for the translation of the RSO, most of the entries appeared to have culturally equivalent terms in Chinese, and we could translate them without further modification. The only exception was N 4: “Getting along with friends around me brings me vitality.” We used the Chinese term “sense of belonging” rather than “getting along” to capture the cultural connotation.

The length and language of the scale were crucial for the elderly to complete the questionnaire, especially for those with limited comprehension and energy. The Chinese version of the RSO had nine items, which were relatively short, simple, and easy to understand. Its content was closely related to the psychological condition of the elderly, making it easier for the elderly to understand. Therefore, the present study has concluded that this scale not only has obvious advantages in terms of statistical indicators, but it can also be more applicable to the social characteristics of the elderly, such as interaction characteristics, language habits, and literacy, while also being more suitable as a tool for measuring the psychological resilience of the elderly in China. During the questionnaire distribution for the original scale, the participants received a questionnaire *via* mail. Considering the reason that the older people were not comfortable filling out the questionnaire on their own because of their visual condition or literacy level, we used face-to-face interviews for questionnaire collection on an item-by-item basis. However, at the same time, care was taken to avoid implicit instruction by the investigator during the item-by-item process.

The RSO may be a useful tool in future epidemiological studies, healthcare, and clinical practice. The tool can allow healthcare professionals to measure the psychological resilience of the oldest-old in the community. The application of this tool may provide an objective and quantitative way for Chinese healthcare professionals to assess the psychological resilience of the oldest-old, facilitating the early identification of the psychosocial status of the oldest-old by healthcare professionals and providing a reliable basis for the later clinical development of interventions for psychological resilience, which has some practical value.

Researchers have confirmed that psychological resilience is a dynamic process (Windle et al., 2008). Therefore, in the future, researchers should understand the level of psychological resilience of older people at different stages (young, old, and oldest-old), which can help enrich the connotation of psychological resilience, strengthen the research on the mechanism of psychological resilience in older people and explore the novel theoretical model of psychological resilience in older people, which will provide a theoretical framework for intervention studies.

The American Psychological Association (APA) has emphasized that training methods for psychological resilience are flexible and varied (Hu et al., 2020). The key to improving resilience is to select the most appropriate method and practice it according to the physical and mental characteristics of the target population. However, there is a paucity of research on psychological resilience interventions for the oldest-old, and there is a wide variation in intervention approaches and a lack of targeting. Therefore, future research should use psychological resilience as an entry point to build an intervention model and conduct in-depth intervention studies on the psychological resilience of the oldest-old to provide creative ideas for promoting healthy aging.

Some limitations need to be addressed. The present study only used a convenience sampling method to select community elders in Shenyang as the study population, so it has certain geographical limitations, the representativeness needs to be improved, and the generalizability of the study results needs to be further verified. It is recommended that future studies further validate and improve the Chinese RSO scale. Considering that China is a multiethnic country with a large population, it is recommended that a multilevel and multicenter study be conducted using a random sampling method, that the sample size is further expanded to improve the reliability and applicability of the scale assessment and that it be revised continuously to make it mature. The current study is based on the Classical Theory Test (CTT), and future studies should use a combination of the Classical Theory Test (CTT) and Item Response Theory (IRT) to provide additional information, such as item difficulty and discrimination, which will help address the factor-related issues in this study (Ye et al., 2018b, 2019; Liang et al., 2021). A limitation of the current study is that no precise distinction was made as to whether the RSO was a state or trait scale (Ye et al., 2020). Therefore, future research should be conducted to sort out the state and trait components of psychological resilience in the oldest-old. To help determine the dynamic goals of a resilience-based intervention trial involving the oldest-old. Finally, further estimates of minimal clinically important differences are needed to facilitate its clinical use (Li and Ow, 2022).

Based on the overall results, the Chinese version of the RSO is a valid and reliable tool that can be used in communities in China to assess resilience in the oldest-old.

## Conclusion

The Chinese version of the RSO showed high reliability with the same one-factor structure used in previous research. The Chinese version of the RSO is a useful measure that could promote the assessment and research on the mental resilience of the elderly in the Chinese population.

## Data availability statement

The raw data supporting the conclusions of this article will be made available by the authors, without undue reservation.

## Ethics statement

The studies involving human participants were reviewed and approved by the Institutional Review Board of the First Hospital of China Medical University. The patients/participants provided their written informed consent to participate in this study.

## Author contributions

WN, CH, and LY were responsible for study conception and design. All authors were responsible for acquisition of data, analysis, and interpretation of data, involved in drafting the manuscript or revising it critically for important intellectual content, and made substantial contributions to this manuscript.

## Conflict of interest

The authors declare that the research was conducted in the absence of any commercial or financial relationships that could be construed as a potential conflict of interest.

## Publisher’s note

All claims expressed in this article are solely those of the authors and do not necessarily represent those of their affiliated organizations, or those of the publisher, the editors and the reviewers. Any product that may be evaluated in this article, or claim that may be made by its manufacturer, is not guaranteed or endorsed by the publisher.

## References

[ref1] Aaronson N. Alonso J. Burnam A. Lohr K. N. Patrick D. L. Perrin E.. (2002). Assessing health status and quality-of-life instruments: attributes and review criteria. Qual. Life Res. 11, 193–205. doi: 10.1023/a:1015291021312 12074258

[ref2] Akatsuka E. Tadaka E. (2021). Development of a resilience scale for oldest-old age (RSO). BMC Geriatr. 21:174. doi: 10.1186/s12877-021-02036-w, PMID: 33691635 PMC7944912

[ref3] Almanasreh E. Moles R. Chen T. F. (2019). Evaluation of methods used for estimating content validity. Res. Social Adm. Pharm. 15, 214–221. doi: 10.1016/j.sapharm.2018.03.066 29606610

[ref4] Alvariza A. Holm M. Benkel I. Norinder M. Ewing G. Grande G.. (2018). A person-centred approach in nursing: validity and reliability of the Carer support needs assessment tool. Eur. J. Oncol. Nurs. 35, 1–8. doi: 10.1016/j.ejon.2018.04.005, PMID: 30057075

[ref5] Browne-Yung K. Walker R. B. Luszcz M. A. (2017). An examination of resilience and coping in the oldest old using life narrative method. Gerontologist 57, gnv137–gnv291. doi: 10.1093/geront/gnv137, PMID: 26511273

[ref6] Campbell-Sills L. Stein M. B. (2007). Psychometric analysis and refinement of the Connor-davidson resilience scale (CD-RISC): validation of a 10-item measure of resilience. J. Trauma. Stress. 20, 1019–1028. doi: 10.1002/jts.20271, PMID: 18157881

[ref7] Chen W. Xie E. Tian X. Zhang G. (2020). Psychometric properties of the Chinese version of the resilience scale (RS-14): preliminary results. PLoS One 15:e0241606. doi: 10.1371/journal.pone.0241606, PMID: 33125417 PMC7598507

[ref8] Cheng C. Dong D. He J. Zhong X. Yao S. (2020). Psychometric properties of the 10-item Connor-Davidson resilience scale (CD-RISC-10) in Chinese undergraduates and depressive patients. J. Affect. Disord. 261, 211–220. doi: 10.1016/j.jad.2019.10.018, PMID: 31654919

[ref9] Chmitorz A. Kunzler A. Helmreich I. Tüscher O. Kalisch R. Kubiak T.. (2018). Intervention studies to foster resilience - a systematic review and proposal for a resilience framework in future intervention studies. Clin. Psychol. Rev. 59, 78–100. doi: 10.1016/j.cpr.2017.11.002, PMID: 29167029

[ref10] Choi S. W. Gibbons L. E. Crane P. K. (2011). Lordif: an R package for detecting differential item functioning using iterative hybrid ordinal logistic regression/item response theory and Monte Carlo simulations. J. Stat. Softw. 39, 1–30. doi: 10.18637/jss.v039.i08, PMID: 21572908 PMC3093114

[ref11] Clark L. A. Watson D. (2019). Constructing validity: new developments in creating objective measuring instruments. Psychol. Assess. 31, 1412–1427. doi: 10.1037/pas0000626, PMID: 30896212 PMC6754793

[ref12] Connor K. M. Davidson J. R. (2003). Development of a new resilience scale: the Connor-Davidson resilience scale (CD-RISC). Depress. Anxiety 18, 76–82. doi: 10.1002/da.10113 12964174

[ref13] Cosco T. D. Howse K. Brayne C. (2017). Healthy aging, resilience and scale-level. Epidemiol. Psychiatr. Sci. 26, 579–583. doi: 10.1017/s2045796017000324, PMID: 28679453 PMC6998987

[ref14] Cosco T. D. Kaushal A. Richards M. Kuh D. Stafford M. (2016). Resilience measurement in later life: a systematic review and psychometric analysis. Health Qual. Life Outcomes 14:16. doi: 10.1186/s12955-016-0418-6, PMID: 26821587 PMC4730639

[ref15] Fang E. F. Xie C. Schenkel J. A. Wu C. Long Q. Cui H.. (2020). A research agenda for aging in China in the 21st century (2nd edition): focusing on basic and translational research, long-term care, policy and social networks. Aging Res. Rev. 64:101174. doi: 10.1016/j.arr.2020.101174, PMID: 32971255 PMC7505078

[ref16] Feenstra M. Smidt N. van Munster B. C. Glynn N. W. de Rooij S. E. (2020). Translation and validation of the Dutch Pittsburgh fatigability scale for older adults. BMC Geriatr. 20:234. doi: 10.1186/s12877-020-01630-8, PMID: 32641002 PMC7346360

[ref17] Fontes A. P. Neri A. L. (2015). Resilience in aging: literature review. Cien. Saude Colet. 20, 1475–1495. doi: 10.1590/1413-81232015205.00502014 26017950

[ref18] Gao Y. Yuan L. Pan B. Wang L. (2019). Resilience and associated factors among Chinese patients diagnosed with oral cancer. BMC Cancer 19:447. doi: 10.1186/s12885-019-5679-0, PMID: 31088400 PMC6518694

[ref19] Garcia J. M. Gallagher M. W. O'Bryant S. E. Medina L. D. (2021). Differential item functioning of the Beck anxiety inventory in a rural, multi-ethnic cohort. J. Affect. Disord. 293, 36–42. doi: 10.1016/j.jad.2021.06.005, PMID: 34166907 PMC8349838

[ref21] Hardy S. E. Concato J. Gill T. M. (2004). Resilience of community-dwelling older persons. J. Am. Geriatr. Soc. 52, 257–262. doi: 10.1111/j.1532-5415.2004.52065.x 14728637

[ref22] Hayman K. J. Kerse N. Consedine N. S. (2017). Resilience in context: the special case of advanced age. Aging Ment. Health 21, 577–585. doi: 10.1080/13607863.2016.1196336, PMID: 27333589

[ref23] Hervé F. Ragolle I. Amarenco G. Viaene A. Guinet-Lacoste A. Bonniaud V.. (2019). Assessment of intermittent self-catheterization procedures in patients with neurogenic lower urinary tract dysfunction: Dutch translation and validation of the intermittent catheterization satisfaction questionnaire, intermittent catheterization acceptance test, intermittent self catheterization questionnaire and intermittent catheterization difficulty questionnaire. Urol. Int. 102, 476–481. doi: 10.1159/000499884, PMID: 30999304

[ref24] Ho H. Y. Lee Y. L. Hu W. Y. (2012). Elder resilience: a concept analysis. Hu Li Za Zhi 59, 88–92. doi: 10.1891/9780826126825.0002 22469896

[ref25] Hodiamont F. Hock H. Ellis-Smith C. Evans C. de Wolf-Linder S. Jünger S.. (2021). Culture in the spotlight-cultural adaptation and content validity of the integrated palliative care outcome scale for dementia: a cognitive interview study. Palliat. Med. 35, 962–971. doi: 10.1177/02692163211004403, PMID: 33863246 PMC8114430

[ref26] Hu C. Chung P. K. Zhang C. Q. Gan Y. Hu R. (2020). Understanding of resilience of older adults in Hong Kong: a qualitative investigation. Gerontol. Geriatr. Med. 6:6904. doi: 10.1177/2333721420966904, PMID: 33195741 PMC7597569

[ref27] Itzhaki M. Peles-Bortz A. Kostistky H. Barnoy D. Filshtinsky V. Bluvstein I. (2015). Exposure of mental health nurses to violence associated with job stress, life satisfaction, staff resilience, and post-traumatic growth. Int. J. Ment. Health Nurs. 24, 403–412. doi: 10.1111/inm.12151, PMID: 26257307

[ref28] Jones R. N. (2019). Differential item functioning and its relevance to epidemiology. Curr Epidemiol Rep 6, 174–183. doi: 10.1007/s40471-019-00194-5, PMID: 31840016 PMC6910650

[ref29] Ju J. Jiang Y. Zhou P. Li L. Ye X. Wu H.. (2018). Evaluation of the reliability and validity for X16 balance testing scale for the elderly. BMC Geriatr. 18:112. doi: 10.1186/s12877-018-0803-6, PMID: 29807543 PMC5971429

[ref30] Kalisch R. Müller M. B. Tüscher O. (2015). A conceptual framework for the neurobiological study of resilience. Behav. Brain Sci. 38:e92. doi: 10.1017/s0140525x1400082x, PMID: 25158686

[ref31] Li Y. T. Ow Y. S. Y. (2022). Development of resilience scale for older adults. Aging Ment. Health 26, 159–168. doi: 10.1080/13607863.2020.1861212 33410343

[ref32] Liang M. Z. Tang Y. Chen P. Liang J. Sun Z. Hu G. Y.. (2021). New resilience instrument for family caregivers in cancer: a multidimensional item response theory analysis. Health Qual. Life Outcomes 19:258. doi: 10.1186/s12955-021-01893-8, PMID: 34794439 PMC8600888

[ref33] Luo R. Z. Liu J. Y. Zhang C. M. Liu Y. H. (2021). Chinese version of the clinical supervision self-assessment tool: assessment of reliability and validity. Nurse Educ. Today 98:104734. doi: 10.1016/j.nedt.2020.104734, PMID: 33465678

[ref34] MacLeod S. Musich S. Hawkins K. Alsgaard K. Wicker E. R. (2016). The impact of resilience among older adults. Geriatr. Nurs. 37, 266–272. doi: 10.1016/j.gerinurse.2016.02.014 27055911

[ref35] Manning L. K. Carr D. C. Kail B. L. (2016). Do higher levels of resilience buffer the deleterious impact of chronic illness on disability in later life? Gerontologist 56, 514–524. doi: 10.1093/geront/gnu068, PMID: 25063353 PMC4873762

[ref36] Manning L. Ferris M. Rosario C. N. Prues M. Bouchard L. (2019). Spiritual resilience: understanding the protection and promotion of well-being in the later life. J. Relig. Spiritual. Aging 31, 168–186. doi: 10.1080/15528030.2018.1532859, PMID: 33335455 PMC7743140

[ref37] Manning L. K. Miles A. (2018). Examining the effects of religious attendance on resilience for older adults. J. Relig. Health 57, 191–208. doi: 10.1007/s10943-017-0438-5, PMID: 28744592

[ref38] Marsh H. W. Hau K. T. Balla J. R. Grayson D. (1998). Is more ever too much? The number of indicators per factor in confirmatory factor analysis. Multivar. Behav. Res. 33, 181–220. doi: 10.1207/s15327906mbr3302_1, PMID: 26771883

[ref39] Polit D. F. Beck C. T. (2006). The content validity index: are you sure you know what's being reported? Critique and recommendations. Res. Nurs. Health 29, 489–497. doi: 10.1002/nur.20147, PMID: 16977646

[ref40] Prosser S. J. Metzger M. Gulbransen K. (2017). Don't just survive, thrive: understanding how acute psychiatric nurses develop resilience. Arch. Psychiatr. Nurs. 31, 171–176. doi: 10.1016/j.apnu.2016.09.010, PMID: 28359429

[ref41] R Core Team. (2016). R: A language and environment for statistical computing. Vienna, Austria: R Foundation for Statistical Computing. Available at: https://www.rproject.org

[ref42] Rezaeipandari H. Mohammadpoorasl A. Morowatisharifabad M. A. Shaghaghi A. (2022). Psychometric properties of the Persian version of abridged Connor-Davidson resilience scale 10 (CD-RISC-10) among older adults. BMC Psychiatry 22:493. doi: 10.1186/s12888-022-04138-0, PMID: 35869455 PMC9308300

[ref43] Shi X. Wang S. Wang Z. Fan F. (2021). The resilience scale: factorial structure, reliability, validity, and parenting-related factors among disaster-exposed adolescents. BMC Psychiatry 21:145. doi: 10.1186/s12888-021-03153-x, PMID: 33691656 PMC7945311

[ref44] Siltanen S. Tourunen A. Saajanaho M. Palmberg L. Portegijs E. Rantanen T. (2021). Psychological resilience and active aging among older people with mobility limitations. Eur. J. Aging 18, 65–74. doi: 10.1007/s10433-020-00569-4, PMID: 33746682 PMC7925737

[ref45] Sinclair V. G. Wallston K. A. (2004). The development and psychometric evaluation of the brief resilient coping scale. Assessment 11, 94–101. doi: 10.1177/1073191103258144, PMID: 14994958

[ref48] Tay P. K. C. Lim K. K. (2020). Psychological resilience as an emergent characteristic for well-being: a pragmatic view. Gerontology 66, 476–483. doi: 10.1159/000509210, PMID: 32784301

[ref49] Terwee C. B. Bot S. D. de Boer M. R. van der Windt D. A. Knol D. L. Dekker J.. (2007). Quality criteria were proposed for measurement properties of health status questionnaires. J. Clin. Epidemiol. 60, 34–42. doi: 10.1016/j.jclinepi.2006.03.012 17161752

[ref50] Tian X. Lu J. Che Y. Fang D. Ran H. He X.. (2021). Childhood maltreatment and self-harm in Chinese adolescents: moderation and mediation via resilience. BMC Public Health, 21:1561. doi: 10.1186/s12889-021-11605-y, PMID: 34404376 PMC8371889

[ref51] Tomas J. M. Caycho-Rodríguez T. Ventura-León J. Sancho P. García C. H. Arias W. L. (2021). Measurement invariance of the brief resilient coping scale (BRCS) in Peruvian and Spanish older adults. J. Cross Cult. Gerontol. 36, 431–444. doi: 10.1007/s10823-021-09441-z, PMID: 34748118 PMC8591005

[ref52] Wagnild G. M. Young H. M. (1993). Development and psychometric evaluation of the resilience scale. J. Nurs. Meas. 1, 165–178. 7850498

[ref53] Wermelinger Ávila M. P. Lucchetti A. L. Lucchetti G. (2017). Association between depression and resilience in older adults: a systematic review and meta-analysis. Int. J. Geriatr. Psychiatry 32, 237–246. doi: 10.1002/gps.4619, PMID: 27805730

[ref54] Windle G. Markland D. A. Woods R. T. (2008). Examination of a theoretical model of psychological resilience in older age. Aging Ment. Health 12, 285–292. doi: 10.1080/13607860802120763, PMID: 18728940

[ref55] Wu L. Tan Y. Liu Y. (2017). Factor structure and psychometric evaluation of the Connor-Davidson resilience scale in a new employee population of China. BMC Psychiatry 17:49. doi: 10.1186/s12888-017-1219-0, PMID: 28152997 PMC5290619

[ref56] Xie Y. Peng L. Zuo X. Li M. (2016). The psychometric evaluation of the Connor-Davidson resilience scale using a Chinese military sample. PLoS One 11:e0148843. doi: 10.1371/journal.pone.0148843, PMID: 26859484 PMC4747584

[ref57] Xu X. Zhao Y. Zhou J. Xia S. (2022). Quality-of-life evaluation among the oldest-old in China under the "active aging framework". Int. J. Environ. Res. Public Health 19:4572. doi: 10.3390/ijerph19084572, PMID: 35457438 PMC9031229

[ref58] Yang Y. Wen M. (2015). Psychological resilience and the onset of activity of daily living disability among older adults in China: a Nationwide longitudinal analysis. J. Gerontol. B Psychol. Sci. Soc. Sci. 70, 470–480. doi: 10.1093/geronb/gbu068, PMID: 24898031

[ref59] Ye Z. J. Liang M. Z. Li P. F. Sun Z. Chen P. Hu G. Y.. (2018a). New resilience instrument for patients with cancer. Qual. Life Res. 27, 355–365. doi: 10.1007/s11136-017-1736-9, PMID: 29119454

[ref60] Ye Z. J. Liang M. Z. Zhang H. W. Li P. F. Ouyang X. R. Yu Y. L.. (2018b). Psychometric properties of the Chinese version of resilience scale specific to cancer: an item response theory analysis. Qual. Life Res. 27, 1635–1645. doi: 10.1007/s11136-018-1835-2, PMID: 29569015

[ref61] Ye Z. J. Zhang Z. Tang Y. Liang J. Sun Z. Zhang X. Y.. (2019). Development and psychometric analysis of the 10-item resilience scale specific to cancer: a multidimensional item response theory analysis. Eur. J. Oncol. Nurs. 41, 64–71. doi: 10.1016/j.ejon.2019.06.005, PMID: 31358259

[ref62] Ye Z. J. Zhang Z. Zhang X. Y. Tang Y. Chen P. Liang M. Z.. (2020). State or trait? Measuring resilience by generalisability theory in breast cancer. Eur. J. Oncol. Nurs., 46:101727. doi: 10.1016/j.ejon.2020.101727 32339909

[ref63] Zhang J. Yu N. X. Zhou M. Zhang J. (2017). Dyadic effects of resilience on well-being in Chinese older couples: mediating role of spousal support. J. Fam. Psychol. 31, 273–281. doi: 10.1037/fam0000250, PMID: 27690500

[ref64] Zhou S. Zhao Q. Weng H. Wang N. Wu X. Li X.. (2021). Translation, cultural adaptation and validation of the Chinese version of the Carer support needs assessment tool for family caregivers of cancer patients receiving home-based hospice care. BMC Palliat. Care 20:71. doi: 10.1186/s12904-021-00766-7, PMID: 34011333 PMC8136129

